# Elevated Troponin and Jarisch-Herxheimer Reaction in Tick-Borne Relapsing Fever: A Case Report

**DOI:** 10.1155/2011/950314

**Published:** 2011-10-09

**Authors:** Kenneth A. Hoekstra, Michael T. Kelly

**Affiliations:** ^1^Immunology Department, Peace Health Laboratories, 2901 Squalicum Parkway, Bellingham, WA 98225, USA; ^2^Microbiology and Immunology Departments, Peace Health Laboratories, Bellingham, WA 98225, USA

## Abstract

We describe a patient with fever and borreliosis in the Northwestern Washington State, USA. The patient exhibited a classic Jarisch-Herxheimer reaction of tachycardia, hypotension, and thrombocytopenia following antimicrobial therapy, and she also developed an elevated serum cardiac troponin during therapy.

## 1. Introduction

 Tick-borne relapsing fever is caused by many different *Borrelia* species. One soft-bodied tick, *Ornithodoros hermsii* acts as a vector for the organism *B. hermsii* [1]. These ticks are common in the Western United States and Southern British Columbia [2]. The spirochete-infected ticks feed for short periods during the night and then fall off. During the ensuing spirochetemia, a sudden onset of febrile illness occurs and is characterized by rigors, headache, myalgia, vomiting, abdominal pain, and cough [3, 4]. Definitive diagnosis can be made by the microscopic identification of loosely coiled spirochetes in a peripheral blood smear. We report a case of borreliosis in Whatcom County, Wash, USA complicated by the Jarisch-Herxheimer reaction (J-HR) and increased cardiac troponin. 

## 2. Case Report

**Figure 1:**
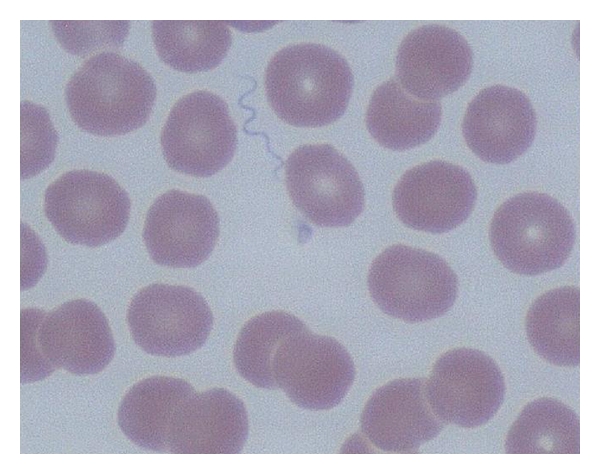
Loosely coiled *Borrelia* sp. in a thin peripheral blood smear stained with Wright-Giemsa stain and visualized under oil immersion (×500).

**Figure 2:**
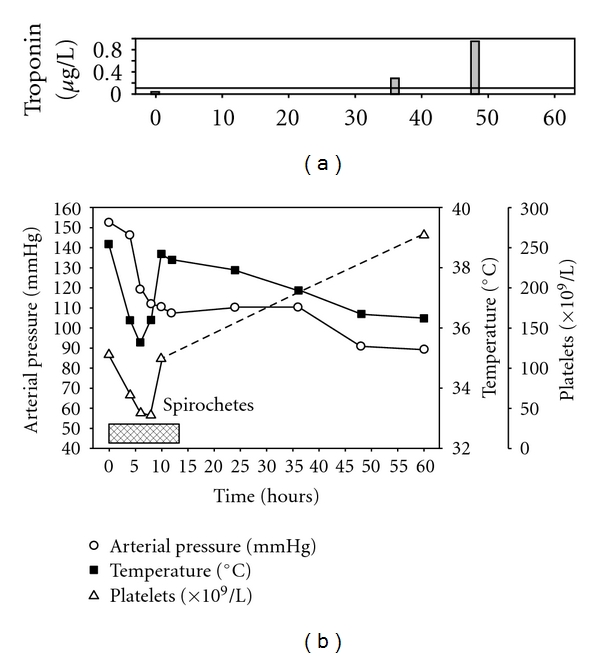
Time course of Jarisch-Herxheimer reaction (J-HR) in relapsing fever. Antimicrobial therapy was started at time 0. Symptoms of rapid decrease in temperature, hypotension, and thrombocytopenia correlated with peripheral blood spirochete clearance (pattern). Sepsis resulted in elevated serum cardiac troponin without acute coronary syndrome at 36 and 48 hours compared to time 0.

A 75-year-old woman was seen at a local hospital with a chief complaint of febrile illness and myalgia. She had a heart rate of 145 bpm and a systolic blood pressure of 140 mmHg (Figure 1). Past medical history was unremarkable. She reported no known drug allergies and no current medications. There was no recent travel history. She reported no known tick bites. No unusual dietary exposure was noted. Within 30 minutes of admission, she complained of muscle cramps and had clammy skin and diarrhea. She appeared dehydrated and was started on saline intravenously for clinical dehydration and diagnosed with a likely viral illness and possible sepsis. A urinalysis revealed 3+ leukocyte esterase, 2+ protein, 95 white blood cells/hpf, and many bacteria. A comprehensive metabolic panel showed elevated renal (urea nitrogen = 10.5 mmol/L, creatinine = 114.9 *μ*mol/L) and liver enzymes (AST = 114 U/L, ALT = 163 U/L), high glucose (8.56 mmol/L), and normal electrolytes. The patient was nonreactive for Hepatitis A IgM, Hepatitis B core IgM and surface IgG antibodies, and Hepatitis B surface antigen and HIV. A baseline troponin I was 0.03 *μ*g/L. A complete blood count showed white blood cells (WBC) of 10.0 × 10^9^/L and a hematocrit of 0.404. She was thrombocytopenic with a platelet count of 92 × 10^9^/L. Microscopic examination of the peripheral blood smear revealed numerous loosely-coiled spirochetes consistent with *Borrelia* sp. infection (Figure 2). A working diagnosis of tick-borne relapsing fever (TBRF) was made. The patient was started on pantoprazole, levofloxin, and acetaminophen. Within 4 hours of antibiotic administration there was a clinical worsening of symptoms complicated by Jarisch-Herxheimer reaction (J-HR). The patient's temperature, blood pressure, and platelet count decreased rapidly (Figure 1). A urine culture was positive for *E. coli*, and antimicrobial therapy was changed to ertapenem and ciprofloxin based on susceptibility testing results. At 12 hours, spirochetes were no longer visible on the peripheral, and the patient became afebrile. At the time of 36 hours of admission, the patient complained of chest and low-back pain. A repeat troponin was 0.28 *μ*g/L. She was started on metoprolol and transferred to the cardiovascular unit for observation. At the time of 48 hrs, a repeat troponin was 0.96 *μ*g/L. During the next 12 hours of supportive treatment, the thrombocytopenia improved (112 × 10^9^/L), and angina and febrile illness resolved. She was discharged with instructions to follow up with her primary care provider within 10 days. Fourteen days after onset of the illness, the patient's platelet count had normalized to 235 × 10^9^/L. Serological testing for *Borrelia* sp. was performed on specimens collected on day 1 (acute) and on day 120 (convalescent). The acute specimen was negative whereas the convalescent specimen was positive for antibodies to *B. hermsi*.

## 3. Discussion

 The J-HR is a well-known complication of antimicrobial therapy to borreliosis. Similar to this case study, Webster et al. (2002) outlined a J-HR following administration of fluoroquinolones in TBRF [5]. Other studies have shown that cytokines, namely, tumor necrosis factor, interleukin-8, and -16, appear in the circulation transiently and correlate with symptom severity in TBRF [6]. Antibodies against inflammatory cytokines have been shown to decrease the J-HR [7]. Sepsis that results from the presence of infectious organisms or their toxins in the blood is frequently associated with changes in these inflammatory mediators [8]. Furthermore, elevations in cardiac troponin in patients with sepsis are common [9]. Notably, this is one of the first published reports of transient increases in cardiac troponin in TBRF. The potential causes of troponin release during sepsis include decreased cardiac membrane integrity, bacterial endotoxins, and thrombotic dysfunction and are unlikely due to flow-limiting etiologies [10]. While the therapy of TBRF is well established and the response to antimicrobial agents is predictably good, complications such as sepsis, the J-HR and elevated cardiac troponin may increase mortality rates.
